# Fecal Microbiota of Diarrhea-Predominant Irritable Bowel Syndrome Patients Causes Hepatic Inflammation of Germ-Free Rats and Berberine Reverses It Partially

**DOI:** 10.1155/2019/4530203

**Published:** 2019-04-03

**Authors:** Qiong Jia, Lu Zhang, Jindong Zhang, Fei Pei, Shiwei Zhu, Qinghua Sun, Liping Duan

**Affiliations:** ^1^Department of Gastroenterology, Peking University Third Hospital, No. 49 North Garden Rd., Haidian District, Beijing 100191, China; ^2^Department of Pathology, Peking University Health Science Center, No. 38 Xueyuan Rd., Haidian District, Beijing 100191, China

## Abstract

Effects of the microbiome associated with diarrhea-predominant irritable bowel syndrome (IBS-D) on the gut have been reported, but no study has reported the effects of the IBS-D gut microbiome on the liver. We transplanted the fecal microbiota from an IBS-D patient and from a healthy volunteer to GF rats. The hepatic inflammation, serum biochemical parameters and metabolome, fecal microbiota profile, fecal short-chain fatty acids (SCFAs), and correlations among them before and after berberine intervention were assessed. Compared with the healthy control fecal microbiome transplantation (FMT) rats, the fecal microbiota of IBS-D patients induces significant Kupffer cell hyperplasia, hepatic sinusoid hypertrophy, and elevated levels of hepatic tumor necrosis factor-*α* and interferon-*γ* and decreases the synthesis of ALB in GF rats. This is possibly related to* Faecalibacterium* and* Bifidobacterium *attributable to fecal formate, acetate, and propionate levels, which are associated with the host linoleic acid pathway. Berberine can partially reverse the Kupffer cell hyperplasia,* Faecalibacterium*, fecal formate, acetate, and propionate by modulating the gut microbiome composition. These results may imply that IBS-D not only is an intestinal functional disorder but can cause liver inflammation, thus providing some implications regarding the clinical cognition and treatment of IBS-D.

## 1. Introduction

Dysbiosis of the gut microbiota has been reported to contribute to diarrhea-predominant irritable bowel syndrome (IBS-D) [1, 2]. Our previous study showed that IBS-D patients have* Bacteroides*-dominant (type I) or* Prevotella*-dominant (type II) gut microbial signatures that are associated with colonic mucosa inflammation; however, healthy controls have a nondominant microbial profile (type III) [3]. Dysbiosis contributes to increased intestinal permeability [4], which allows microbial products to translocate from intestinal lumen to liver. All liver functions, including substrate and energy metabolism, oxidative stress, glycogen storage, and synthesis of secretory proteins, are impacted by liver-microbial interactions [5, 6]. To the best of our knowledge, no study of the effects on the liver and possible mechanism of the fecal microbiome from IBS-D patients has been reported.

Kupffer cells (KCs), also known as hepatic macrophages, have active roles in immune regulation of the liver [7]. Bile acids (BAs) and the gut microbiome modulate each other [8]. By binding with the farnesoid X receptor (FXR; also known as NR1H4), which is a BA receptor, BAs induce the production of antimicrobial peptides, which inhibit gut microbial overgrowth [9]. Microbiota dysbiosis affects the balance between primary and secondary BAs and their enterohepatic cycling [10]. Short-chain fatty acids (SCFAs) are the end products of bacterial fermentation of indigestible dietary components such as plant fiber, and they have a key role in maintaining gut health. However, IBS-D patients have acetate and propionate levels that are significantly higher compared to those of healthy controls [11]. SCFAs can enter the systemic circulation, and they may influence metabolic and immune pathways [12].

Berberine (BBR) is a natural isoquinoline alkaloid that is isolated from the stems and roots of several plants; it is traditionally used to treat enteritis [13]. Recently, studies have revealed that BBR can treat high-fat diet-induced obesity by modulating the gut microbiota and reduce insulin resistance via the Toll-like receptor-4 signaling pathway in rats [14, 15].

The aims of the present study were to identify the effects and possible mechanisms of the fecal microbiota of IBS-D patients on the liver in germ-free (GF) rats and to assess whether BBR could reverse the effects on the liver caused by IBS-D fecal microbiome transplantation (FMT).

## 2. Materials and Methods

### 2.1. Preparation of the Fecal Microbiota from a Patient with IBS-D and a Healthy Control

Fecal samples from a typical IBS-D patient (*Bacteroides*-dominant) and a healthy control (HC, nondominant microbiota), both without any liver diseases [3], were suspended with sterilized phosphate-buffered saline (PBS; with 20% glycerol) 50 times (mass/volume) and transplanted to GF rats by gavage over the course of 1 hour. Rats in the same group received fecal microbiota from the same sample, and transplantation was performed only once.

### 2.2. Animal and Study Protocols

GF Sprague Dawley (SD) rats were purchased from Peking University Health Science Center Experimental Animal Science Center and raised in sterilized isolators at room temperature (25°C) with relative humidity (50%). All materials used during this experiment were sterilized with high-temperature and high-pressure sterilization, 5% peracetic acid, or ethylene oxide. Every rat was kept in a single cage, administered food and water ad libitum, and lived under a strict 12-hour light cycle. The animal experiment was approved by the Animal Care Committee of Peking University Health Science Center (LA2016230).

Twenty-six male GF SD rats (6-7 weeks old) were divided into four groups. The GH group comprised GF rats that underwent FMT with a specimen from the healthy volunteer (n=6). The GI group comprised GF rats that underwent FMT with a sample from the IBS-D patient (n=6). The GIB group comprised GI group rats that were treated with BBR (n=7). The GIV group comprised GI group rats that were treated with sterile water as a control (n=7).

GF rats were acclimatized for 1 week to standard experiment conditions prior to FMT. The fecal microbiota was transplanted into GF rats using 1 mL of suspension per rat. Two weeks later, the livers were collected and immediately stored at −80°C and were fixed in paraformaldehyde. The GIB group received BBR 200 mg/kg and the GIV group received the same volume of diluted water per day for 2 weeks. Then, the livers were collected and stored as described previously (Supplementary Figure S1).

### 2.3. Biochemical Parameters

Blood samples (approximately 5 mL) were collected from the heart and biochemical parameters (including serum alanine aminotransferase [ALT], aspartate aminotransferase [AST], albumin [ALB], lactate dehydrogenase [LDH], *γ*-glutamyl transpeptidase [GGT], and direct bilirubin [DBIL]) were measured using an automatic biochemical analyzer. Fecal lipopolysaccharide (LPS) levels were assessed using an enzyme-linked immunosorbent assay.

### 2.4. Histology and Immunohistochemistry Staining of KCs

After being dehydrated and embedded in paraffin, liver tissues were sectioned and stained with hematoxylin and eosin (HE). Immunohistochemistry was performed with CD68 using standard histological techniques described in the Supplementary Materials. The level of KCs hyperplasia was judged by counting the positive cell numbers of three random high-power fields (×400) per sample. Expansion of hepatic sinusoid and fibrosis was scored as follows: 0, negative; 1, slight; 2, mild; 3, moderate; and 4, strong.

### 2.5. Western Blot Analysis

Liver tissues were homogenized in protein lysis buffer containing protease inhibitor. The protein concentration was determined by using bovine serum albumin as the standard. Proteins were denatured with 5× loading buffer in a heating block for 5 minutes. Equal amounts of whole liver tissue protein extracts were analyzed by SDS-polyacrylamide gel electrophoresis and standard western blotting analysis using antitumor necrosis factor (TNF)-*α*, anti-interferon (IFN)-*γ*, anti-inducible nitric oxide synthase (iNOS), and anti-NR1H4 antibodies (1:500, respectively; Abcam, Cambridge, MA, USA). The level of each protein was normalized to that of the housekeeping gene *β*-actin (1:2000; PPLYGEN, Beijing, China) or GAPDH (1:5000; Abcam) in the same sample using Gel-pro software (Media Cybernetics, LP, MD, USA).

### 2.6. Metabolomics Profiling of Serum

All samples were acquired using a liquid chromatography-mass spectrometry system. All chromatographic separations were performed using an ultraperformance liquid chromatography system (Waters, Manchester, UK). A high-resolution tandem mass spectrometer (Xevo G2 XS Q-TOF; Waters) was used to detect metabolites eluted from the column. The Q-TOF was operated in the positive and negative ion modes. During the positive ion mode, the capillary and sampling cone voltages were set at 3 kV and 40 V, respectively. During the negative ion mode, the capillary and sampling cone voltages were set at 1 kV and 40 V, respectively. The mass spectrometry data were acquired in the Centroid MSE mode.

### 2.7. Microbial DNA Isolation from Rat Feces and 16S rRNA Gene Sequencing

Microbial DNA was isolated from fecal samples collected using the E.Z.N.A. ® DNA Kit (Omega Bio-Tek, Norcross, GA, USA) according to the manufacturer's protocol before the rats were euthanized. The V4-V5 region of the bacterial 16S ribosomal RNA gene was amplified using polymerase chain reaction. Purified amplicons were placed in equimolar pools, and paired-end sequencing was performed on an Illumina MiSeq platform according to the standard protocols.

### 2.8. Fecal SCFAs Assay

Fecal SCFAs, including formate, acetate, propionate, butyrate, isobutyrate, and valerate, were measured using an isotope-labeled chemical derivatization method for liquid chromatography-tandem mass spectrometry with slightly modified parameters. The Dionex Ultimate 3000 UPLC system was coupled to a TSQ Quantiva Ultra triple-quadrupole mass spectrometer (Thermo Fisher, Sunnyvale, CA, USA) equipped with a heated electrospray ionization probe in the negative ion mode. Data were acquired using selected reaction monitoring for each fatty acid. Details are described in the Supplementary Materials.

### 2.9. Statistical Analysis

All data were expressed as the mean ± standard error of the mean (SEM). Comparisons among multiple groups were performed with a nonparametric analysis of variance test, and p<0.05 was considered statistically significant. Correlations between bacterial and metabolic or physiological parameters were tested using Spearman's correlation.

## 3. Results

### 3.1. Effects of IBS-D FMT on Rats

**Figure 1:**
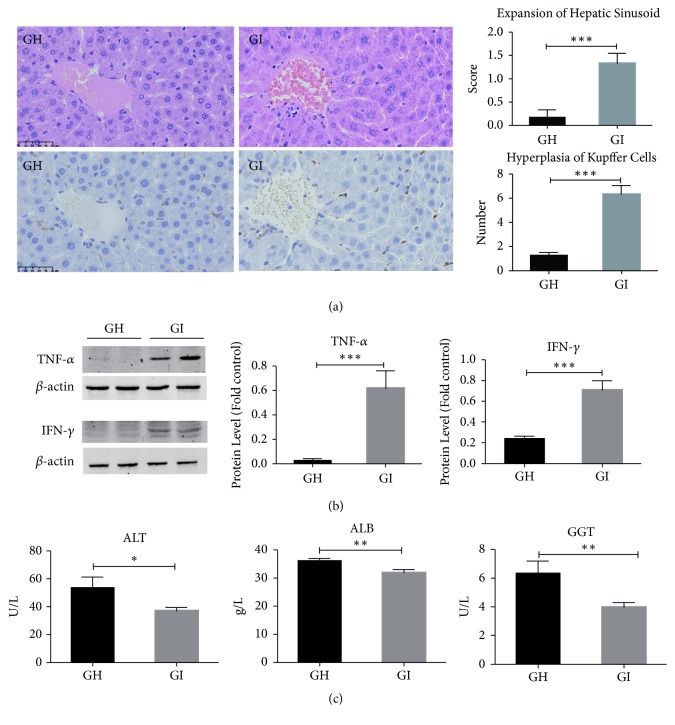
Hepatic inflammation caused by IBS-D. (a) HE and immunohistochemical staining of the liver, hepatic sinusoid expansion score, and number of KCs with hyperplasia. (b) TNF-*α* and IFN-*γ* were examined by western blotting, and each expression was quantified by Gel-pro software. (c) Statistical analysis of serum biochemical parameters. *∗*p<0.05; *∗∗*p<0.01; *∗∗*p<0.001.

The rats received fecal microbiota from the patients with IBS-D (GI group) had significantly higher histology scores for expansion of hepatic sinusoid (p=0.0007) and more KCs with hyperplasia per 400× high-power field (p<0.0001) (Figure 1(a)). We did not find hepatic fibrosis in two weeks of FMT (Supplementary Table S1). Using western blot to detect the expression levels of cytokines in liver tissue, we found that TNF-*α* (p=0.0007) and IFN-*γ* (p=0.0005) levels in the GI group were significantly higher than those in the GH group (Figure 1(b)). Serum ALT (p=0.037), ALB (p=0.005), and GGT (p=0.013) levels of the GI group were significantly lower (Figure 1(c)). Other serum biochemical parameters were not significantly different between the GH and GI groups (Supplementary Table S2). In summary, the fecal microbiome from IBS-D patients significantly induced hepatic inflammation and affected the synthesis of ALT, ALB, and GGT in GF rats.

### 3.2. Characteristics of the Gut Microbiome in the FMT Groups

**Figure 2:**
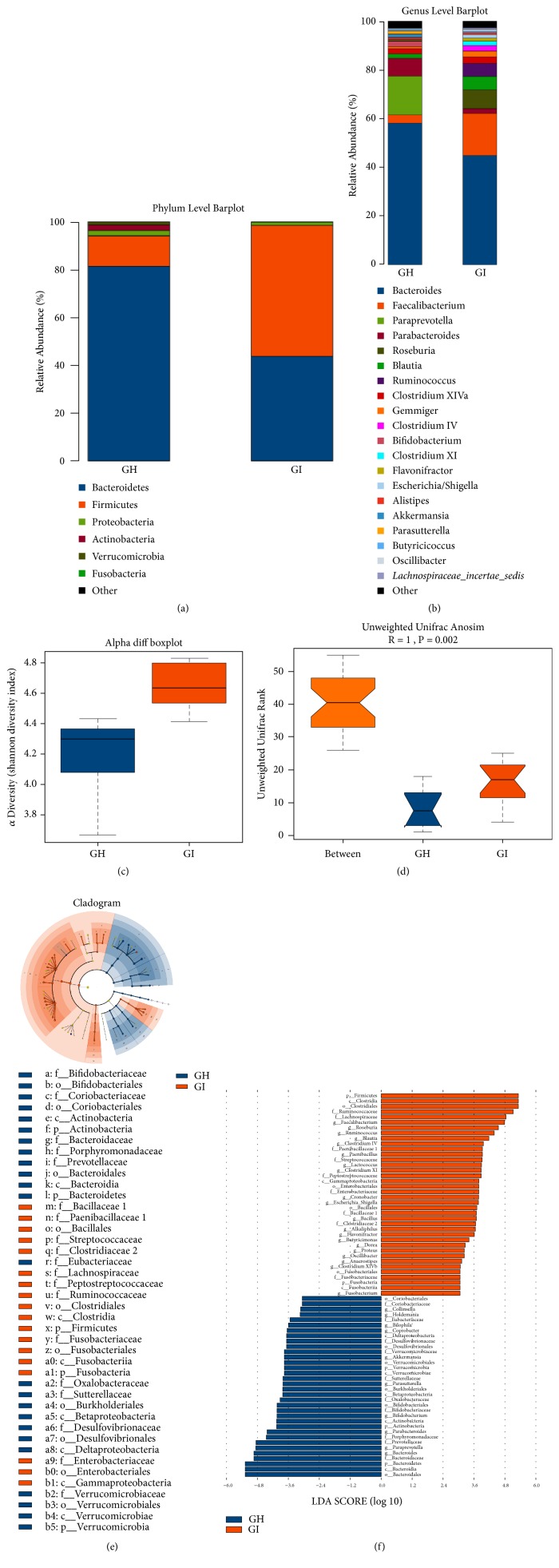
The fecal 16S rRNA sequence after FMT of a sample from an IBS-D patient to GF rats. (a) Difference in the abundance at the phylum level between the GH and GI groups. (b) Difference in the abundance at the genus level between the GH and GI groups. (c) Alpha diversity (Shannon diversity index). (d) Beta diversity (Anosim). Linear discriminant analysis effect size (LefSe) analysis: cladogram (e) and linear discriminant analysis (LDA) (f).

We characterized the gut microbiome of the GH group and GI group. The three most abundant bacteria at the phylum level in the GH group were* Bacteroidetes*,* Firmicutes*, and* Proteobacteria*, whereas those in the GI group were* Firmicutes*,* Bacteroidetes*, and* Proteobacteria *(Figure 2(a)). The ratio of* Firmicutes* to* Bacteroidetes *(F/B) in the GI group was significantly higher than that in the GH group (1.25 vs. 0.16) (Supplementary Table S3). The three most abundant bacteria at the genus level were* Bacteroides*,* Paraprevotella*, and* Parabacteroides* in the GH group, and* Bacteroides*,* Faecalibacterium*, and* Roseburia* in the GI group (Figure 2(b), Table 1).* Bacteroides*,* Bifidobacterium*,* Paraprevotella*, and* Akkermansia* in the GI group were notably decreased, and* Faecalibacterium*,* Lachnospiraceae_incertae_sedis*,* Ruminococcus*,* Blautia*,* Clostridium XI*, and* Roseburia *in the GI group were markedly increased (Table 1). Alpha diversity (p=0.005) and beta diversity (R=1; p=0.002) are shown in Figures 2(c) and 2(d). A cladogram differently exhibited the predominant microbiota in two groups (Figure 2(e)). The discriminative genus was shown by the linear discriminant analysis effect size, which indicated the predominant genus in each group (Figure 2(f)).

### 3.3. Detection of Fecal SCFAs and LPS

**Figure 3:**
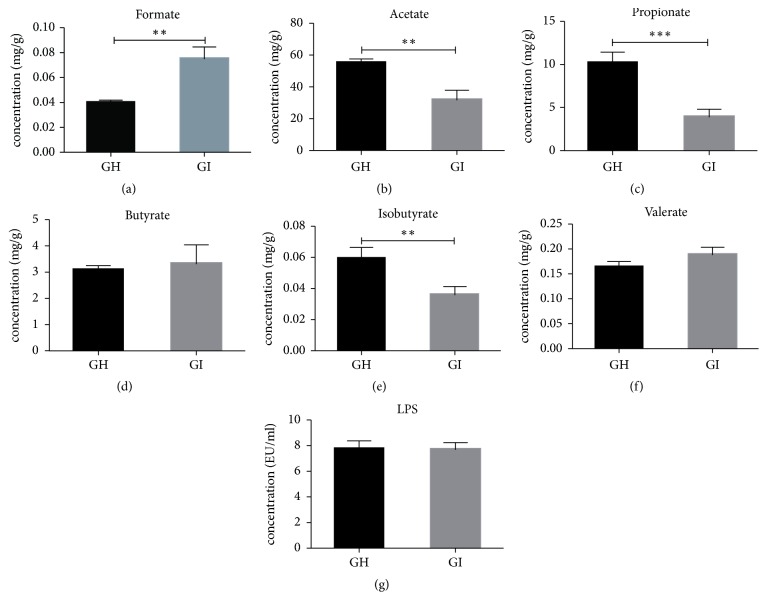
Comparison of fecal SCFAs and LPS levels between the GH and GI groups. (a-f) Formate, acetate, propionate, butyrate, isobutyrate, and valerate. (g) Fecal LPS. *∗*p<0.05; *∗∗*p<0.01; *∗∗*p<0.001.

We quantified the levels of fecal SCFAs and LPS. The fecal formate level of the GI group was noticeably higher than that of the GH group, whereas acetate, propionate, and isobutyrate levels were markedly lower than those of the GH group (Figures 3(a)–3(f), Supplementary Table S4). LPS, also known as endotoxin, is the major component of the cell wall of Gram-negative bacteria. There was no significant difference between the GH group and GI group regarding fecal LPS (p=0.46).

### 3.4. Metabolomics of Serum

**Figure 4:**
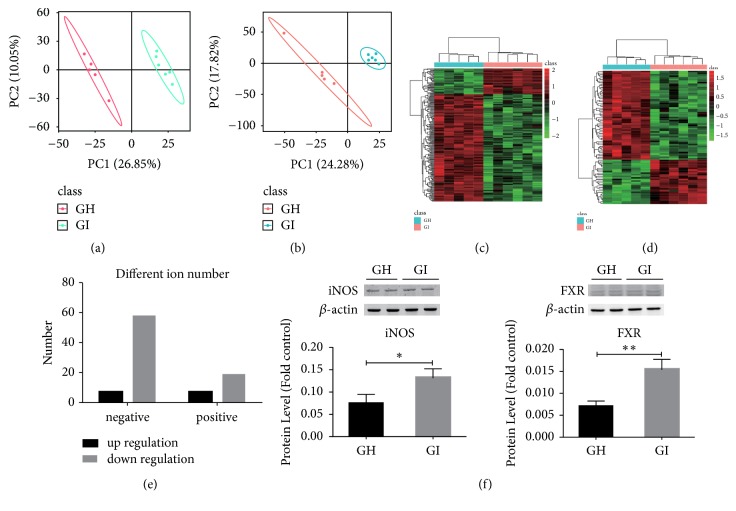
Nontargeted serum metabolomics and expression levels of hepatic iNOS and FXR. (a, b) Partial least-squares discriminant analysis of significantly different negative ions and positive ions. (c, d) Heat maps of significantly different negative ion and positive ion clustering. (e) The number of significantly different negative ions and positive ions in the GI group compared with the GH group. (f) Comparison of liver iNOS and FXR expression levels. *∗*p<0.05; *∗∗*p<0.01.

To investigate the consequences after FMT with different specimens, we performed nontargeted metabolomics profiling of the serum. The GI group had pronounced metabolic alterations compared with the GH group. Ninety-three ions were markedly distinct between the GH and GI groups (p<0.05). Sixty-seven negative ions (seven upregulated and 60 downregulated) and 26 positive ions (seven upregulated and 19 downregulated) were detected in the GI group (Figures 4(a)–4(e)). Altered metabolites were divided into four categories: oxidative stress, BA metabolism, some intermediate materials related to amino acid metabolism, and fatty acid metabolism. Intermediate materials related to fatty acid metabolism were reduced in the GI group. Some intermediate materials related to amino acids, including arginine, proline, aspartate, glutamine, and valine metabolism pathways, were also altered in the GI group. Among them, LL-2, 6-diaminoheptanedioate in the lysine biosynthesis pathway was increased in the GI group; and D-aspartate related to alanine, aspartate, and glutamate metabolism; citraconate related to valine, leucine, and isoleucine biosynthesis; 1-pyrroline-4-hydroxy-2-carboxylate in arginine and proline metabolism; and 5-oxo-D-proline in D-glutamine and D-glutamate metabolism were decreased in the GI group (Supplementary Tables S5 and S6). Two key proteins that were related to oxidative stress and BA metabolism (iNOS and FXR) were quantified by western blotting.  Figure 4(f) revealed that the expression levels of hepatic iNOS (p=0.04) and FXR (p=0.004) in the GI group were increased compared with those in the GH group.

### 3.5. Correlation Analysis

**Figure 5:**
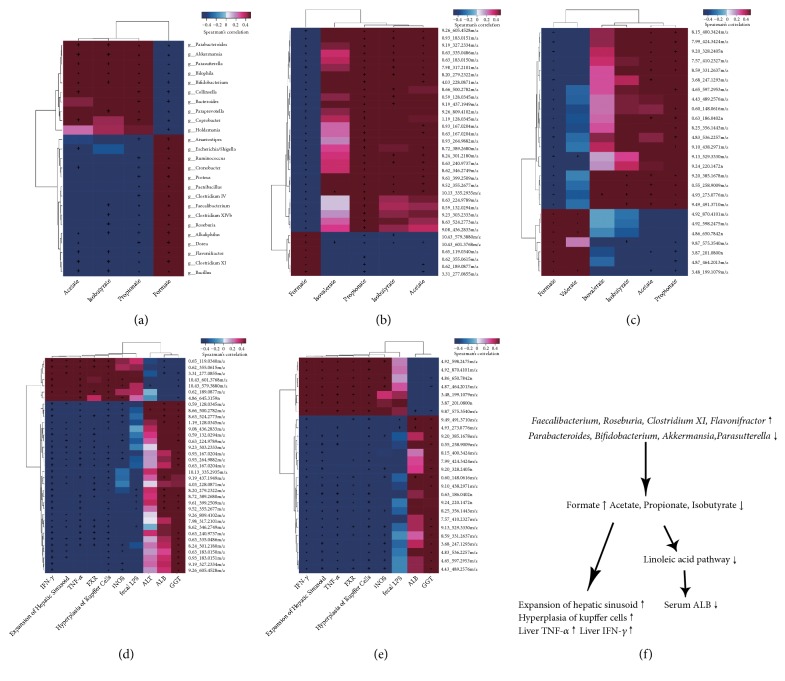
Spearman correlation between the fecal microbiota and fecal SCFAs (a), fecal SCFAs and host metabolites (b, c), and host metabolites and pathophysiological parameters (d, e). (f) The relationship determined from the Spearman correlation analysis.

We performed a bivariate Spearman correlation analysis of fecal microbiota, fecal SCFAs, host metabolites, and pathophysiological parameters of the GH group and GI group. The correlation analysis of the gut microbiota and SCFAs showed that* Faecalibacterium*,* Roseburia*,* Clostridium XI*, and* Flavonifractor*, which were increased in the GI group, were positively related to the fecal formate level but negatively related to the fecal propionate and isobutyrate levels.* Parabacteroides*,* Bifidobacterium*,* Akkermansia*, and* Parasutterella*, which were decreased in the GI group, were negatively related to the level of fecal formate but positively related to the fecal acetate, propionate, and isobutyrate levels (p<0.05) (Figure 5(a)). We also found that the level of fecal formate was positively associated with the increased abundance of host metabolites in the GI group; however, other kinds of SCFAs presented negative correlations (Figures 5(b) and 5(c)). Fecal formate and propionate were associated with hepatic sinusoid expansion, KCs hyperplasia, and protein expression of liver TNF-*α* and IFN-*γ* through most metabolites (Figures 5(b) and 5(e)). Focusing on the correlation between the host metabolites and pathophysiological parameters (Figures 5(d) and 5(e)), we determined that the compound ID 8.20_279.2322 m/z described as linoleic acid, which is involved in linoleic acid metabolism, was significantly positively associated with the serum ALB level (Figure 5(f)). In addition,* Faecalibacterium *and* Bifidobacterium* might affect the synthesis of liver ALB through the host linoleic acid metabolism, thus leading to decreased serum ALB levels. Furthermore, alterations in fecal formate and propionate could lead to liver inflammation.

### 3.6. Intervention of BBR

**Figure 6:**
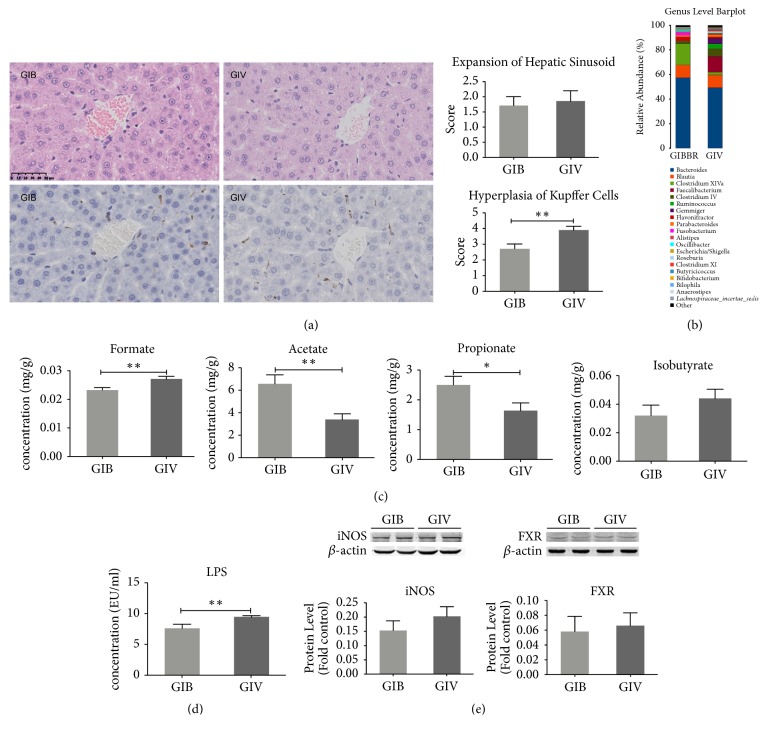
Alteration of the liver and microbiome after BBR intervention. (a) HE and immunohistochemical staining of the liver, score of the hepatic sinusoid expression, and number of KCs with hyperplasia. (b) The altered composition of the fecal microbiome at the genus level after BBR treatment. Quantification of fecal SCFAs (c) and fecal LPS (d). (e) Protein expression levels of hepatic iNOS and FXR. *∗*p<0.05; *∗∗*p<0.01.

KCs hyperplasia in the GIB group was markedly alleviated compared to that in the GIV group (p=0.005) (Figure 6(a)). However, the expression levels of TNF-*α* and IFN-*γ* in liver tissue and the serum ALT, ALB, and GGT levels were not different between those two groups (Supplementary Figure S2a–d). BBR treatment can significantly change the structure profile of the fecal microbiome of the GI group, with a notably decreased F/B ratio at the phylum level (GIB vs. GIV: 0.7 vs. 1.03) and decreased* Bacteroides*,* Faecalibacterium*,* Ruminococcus*,* Gemmiger*,* Roseburia*,* Clostridium XI*, and* Lachnospiraceae_incertae_sedis* at the genus level (Supplementary Table S7, Figure 6(b), Table 1). The fecal formate level was significantly decreased (p=0.005), and acetate (p=0.002) and propionate (GIB: p=0.02) levels were significantly increased compared to the GIV group (Figure 6(c)). Fecal LPS obviously decreased after treatment with BBR (p=0.006) (Figure 6(d)). The expression levels of hepatic iNOS and FXR were downregulated in the GIB group, but not significantly (Figure 6(e)). In conclusion, BBR affected the level of fecal SCFAs by modulating the composition of the gut microbiome to alleviate hepatitis caused by the fecal microbiome of the IBS-D patient.

## 4. Discussion

Pathogenesis of IBS-D involves many respects, including genetic factors, disturbances in the intestinal microbiota, and low-grade mucosal inflammation [1]. Accumulated evidence has already proven that one of the most important developments associated with IBS-D is the alteration of the gut microbiome [3, 16]. Our previous study [3] showed that most IBS-D patients presented significant dysbiosis of gut microbiome which correlated with inflammatory markers of colon tissues. Meanwhile, we found that patients with IBS-D exhibited significant elevation of serum MCP-1 (data not shown) which suggested that patients with IBS-D exhibited systematic inflammation. However, the effects of IBS-D on the gut microbiome and the liver are relatively unknown. The gut-liver axis is a communication system that integrates immunological signals and metabolites with the gut and liver. In this study, we used GF rats to reveal the effects of the IBS-D fecal microbiota on the liver and the effects of BBR intervention and FMT.

Dysbiosis of the gut microbiome disrupts the intestinal epithelial barrier and can result in increased intestinal permeability and translocation of bacteria and their metabolites into the portal vein [5]. The actions of liver damage factors (such as LPS) can stimulate KCs and activate the cell surface receptor Toll-like receptor-4, thus initiating the p38 mitogen-activated protein kinase (MAPK) and nuclear factor *κ*B (NF-*κ*B) signaling pathways, thereby promoting proinflammatory cytokine expression at the transcriptional and translational levels and causing liver injury [17, 18]. We found the fecal microbiota from the IBS-D patient induced significant KCs hyperplasia, hepatic sinusoid hypertrophy, elevated levels of hepatic TNF-*α* and IFN-*γ*, and decreased the synthesis of ALB in GF rats. However, based on the hematoxylin-eosin staining of liver, we did not find hepatic fibrosis in both GH and GI group, which meant that IBS-D fecal microbiota could not induce hepatic fibrosis in two weeks of FMT. All these results suggested that the fecal microbiota of the IBS-D patient could cause systematic inflammation of the liver and affect the synthesis function of ALB. There are some studies that reported no significant difference between fecal and mucosal microbiota of IBS-D patients and HCs and the microbiome are not sufficient to explain the reported altered physiology and symptomatology of IBS-D [19, 20]. We suppose the discrepancy of the features of gut microbiota in patients with IBS-D might be related to two reasons. First, dysbiosis of gut microbiome is one of the pathogenesis of IBS-D. Some patients with IBS show normal gut microbiome. Second, eating habits, mental state, detection methods, and other factors also may lead to different results. However, for our FMT, we chose the* Bacteroides *dominated IBS-D fecal microbiome and nondominant microbiota health control fecal microbiome.

The microbiota of the IBS-D patient had an increased F/B ratio compared with that of the healthy control [21]. The F/B ratio of the GI group was higher than that of the GH group; however, the ratio could be reversed by BBR. Among the altered microbiota,* Faecalibacterium,* which favorably modulates the intestinal immune system, oxidative stress, and colonocyte metabolism, is one of the most abundant and important commensal bacteria of the human gut microbiota [22].* F. prausnitzii*, one species of* Faecalibacterium*, has been shown to secrete anti-inflammatory compounds, such as salicylic acid, in its surrounding environment. Several studies have highlighted that the amount of* F. prausnitzii* negatively correlates with the activity of inflammatory bowel disease and colorectal cancer [23]. We discovered that the abundance of* Faecalibacterium* was dramatically increased after IBS-D FMT and reversed by BBR treatment. This might imply that increasing the abundance of* Faecalibacterium* can decrease the inflammation caused by IBS-D FMT.* Ruminococcus* and* Lachnospiraceae_incertae_sedis* were significantly enriched in chronic hepatitis B patients [24] and nonalcoholic fatty liver disease patients [25], respectively. Furthermore,* Ruminococcus* and* Lachnospiraceae_incertae_sedis *in the GI group in our study were significantly increased compared to those in group GH; however, they were decreased after BBR treatment. This implied that* Ruminococcus* and* Lachnospiraceae_incertae_sedis* have important roles in hepatitis caused by IBS-D FMT. Treatment with BBR also can significantly decrease the abundance of* Gemmiger*,* Roseburia*,* and Clostridium XI. Bifidobacterium* was decreased in hepatocellular carcinoma patients and inversely correlated with calprotectin concentrations, which were associated with humoral and cellular inflammatory markers [26].* Bifidobacterium* was significantly decreased after IBS-D FMT but could not be reversed by BBR treatment. Besides, we noticed that the expression levels of TNF-*α*, IFN-*γ*, and serum ALT, ALB, and GGT in the liver did not change significantly after BBR intervention either; this may have been attributable to the short duration of BBR treatment.

iNOS was originally identified in myeloid cells as a host defense mechanism against pathogens that metabolize L-arginine to produce nitric oxide [27]. Often, iNOS is induced by inflammatory signals. In the current study, we found that serum metabolites in arginine and proline metabolism that are related to iNOS [28] were significantly decreased, but the protein expression level of iNOS was increased in the GI group. These alterations might suggest high availability of these substances in the host and increased antioxidation activity, which may provide protection from oxidative stress and result in decreased serum. FXR-mediated mechanisms prevent the noxious effects of BA accumulation, thus preserving the integrity of the intestinal epithelial barrier and preventing intestinal inflammation [29]. In the liver, FXR has been considered a multifunctional cell protector and tumor suppressor, and it can promote liver regeneration and repair after injury [30]. Hepatic FXR upregulated the protein levels in the IBS-D FMT group and can be reduced by BBR intervention. This suggests that increasing FXR can help protect the host against inflammation caused by the fecal microbiota associated with IBS-D.

A disturbed pathway of synthesis of branched-chain amino acids (BCAAs) has been found in many liver-related diseases [31, 32]. Furthermore, BCAAs are correlated with hypoalbuminemia [33]. Decreased levels of BCAAs were observed during progression regardless of the cause of chronic liver disease [34]. In animal studies, valine, isoleucine, and alanine were increased in the liver of mice with major depressive disorder [35]. Our research by analysis of serum metabolism showed that BCAAs related metabolic pathways were increased, which implied that the gut microbiota associated with IBS-D also included hepatotoxicity.

Spearman correlation analyses implied that the abundance of* Faecalibacterium *and* Bifidobacterium *changed and that there were alterations in the concentrations of fecal formate, acetate, propionate, and isobutyrate, thus leading to hepatic inflammation. Therefore, the microbiota might affect the synthesis of liver ALB through the linoleic acid metabolism of the host, thereby decreasing serum ALB levels and hepatitis. Linolenic acid is an essential fatty acid that is required for a healthy metabolism, and it has been reported to be beneficial in diabetes [36], cancer [37], and heart disease [38]. Molecular interaction studies showed that linolenic acid interacts with high affinity with arginine residues of albumin [39]. Glycation of ALB results in decreased *α*-helical content and alters drug-binding capabilities, and linolenic acid can exert antiglycation activity. As a result, a decrease in linolenic acid caused by the changing composition of gut bacteria was related to the decrease in serum ALB. A decrease in linolenic acid can increase the glycans of ALB and decrease the serum ALB level.

IBS-D as a functional gastrointestinal disorder shows significant dysbiosis of gut microbiome and the dysbiosis can induce liver inflammation of GF rats. These results suggest that dysbiosis of gut microbiome in IBS-D needs more concern and to be evaluated by clinical doctors in order to reduce the impacts to patients. Meanwhile, BBR is a potential drug which can regulate the gut microbiome. Therefore, our study provides new information regarding pathogenesis and management of IBS-D. However, our study had some limitations. We checked the liver only in two weeks of FMT and the long-term impacts of gut dysbiosis in hepatic morphology and function needs further evaluation. Also, we will focus on the possible mechanisms and potential genera of the microbiota to analyze and confirm our results in a future study.

## 5. Conclusions

Our findings demonstrated that the IBS-D gut microbiome can cause significant KCs hyperplasia, hepatic sinusoid hypertrophy, elevated levels of hepatic TNF-*α* and IFN-*γ*, and decrease in serum ALT, ALB, and GGT levels of GF rats; this is possibly related to the increased abundance of* Faecalibacterium* and decreased abundance of* Bifidobacterium*, which lead to alterations in the levels of fecal formate, acetate, and propionate. These changes further affect the linoleic acid metabolism of the host. BBR can significantly reverse KCs hyperplasia, fecal formate, acetate, and propionate by modulating the composition of the gut microbiota. These results may imply that IBS-D not only is an intestinal functional disorder but also can induce liver inflammation. Further, they provide new information regarding pathogenesis and management of IBS-D and raise enough concern about dysbiosis of gut microbiome in IBS-D.

## Figures and Tables

**Table 1:** Significant differences in the abundance at the genus level for the GH and GI groups and for the GIB and GIV groups (p<0.05).

Tax name	GH	GI	Tax name	GIB	GIV
*Faecalibacterium*	3.38	17.20	*Faecalibacterium*	0.24	12.78
*Roseburia*	0.00	7.76	*Roseburia*	0.00	1.28
*Blautia*	1.77	5.46	*Clostridium IV*	1.30	6.25
*Ruminococcus*	0.00	5.41	*Ruminococcus*	0.48	4.10
*Gemmiger*	0.78	2.35	*Gemmiger*	0.01	4.36
*Clostridium XI*	0.14	1.81	*Clostridium XI*	0.00	1.15
*Flavonifractor*	0.40	1.28	*Butyricicoccus*	0.08	0.89
*Escherichia/Shigella*	0.19	1.45	*Clostridium XlVa*	17.40	2.24
*Oscillibacter*	0.40	0.73	*Oscillibacter*	1.12	0.28
*Lachnospiraceae_incertae_sedis*	0.15	0.66	*Lachnospiraceae_incertae_sedis*	0.03	0.49
*Bacteroides*	58.15	44.86	*Bacteroides*	57.38	49.25
*Paraprevotella*	15.86	0.00	*Fusobacterium*	2.00	0.01
*Parabacteroides*	7.41	2.01	*Bilophila*	0.84	0.00
*Bifidobacterium*	2.23	0.02	*Bifidobacterium*	0.00	0.84
*Akkermansia*	1.34	0.00	*Flavonifractor*	3.22	1.12
*Parasutterella*	1.32	0.00	*Anaerostipes*	0.51	0.08

## Data Availability

All data used to support the findings of this study are included within the article except the metabonomics data and parts of the 16S rDNA sequencing data. The metabonomics data and parts of the 16S rDNA sequencing data used to support the findings of this study are included within the supplementary tables.

## Supplementary Materials

Supplementary Materials
*Supplementary Materials and Methods:* histology and immunohistochemistry staining of KCs and assay of short-chain fatty acids.* Supplementary Figure S1:* flowchart of animal experiment.* Supplementary Figure S2:* inflammatory factor and serum biochemical parameters of the liver after BBR intervention.* Supplementary Table S1:* hepatic pathology measurements.* Supplementary Table S2:* clinical measurements.* Supplementary Table S3:* different abundance in phylum level of GH group and GI group.* Supplementary Table S4:* quantification of fecal SCFAs.* Supplementary Tables S5 and S6:* GI/GH group significant different quantified identification filtering negative ions and positive ions, respectively.* Supplementary Table S7:* different abundance in phylum level of GIB group and GIV group.
