# Supplementary material for: Fecal Microbiota of Diarrhea-Predominant Irritable Bowel Syndrome Patients Causes Hepatic Inflammation of Germ-Free Rats and Berberine Reverses It Partially
Source: Biomed Res Int. 2019 Apr 3;2019:4530203. doi: 10.1155/2019/4530203 (PMC6470425; doi:10.1155/2019/4530203)
Supplement: Supplementary Materials — and Methods: histology and immunohistochemistry staining of KCs and assay of short-chain fatty acids. Supplementary Figure S1: flowchart of animal experiment. Supplementary Figure S2: inflammatory factor and serum biochemical parameters of the liver after BBR intervention. Supplementary Table S1: hepatic pathology measurements. Supplementary Table S2: clinical measurements. Supplementary Table S3: different abundance in phylum level of GH group and GI group. Supplementary Table S4: quantification of fecal SCFAs. Supplementary Tables S5 and S6: GI/GH group significant different quantified identification filtering negative ions and positive ions, respectively. Supplementary Table S7: different abundance in phylum level of GIB group and GIV group. [file 4530203.f1.zip › s/Supplementary Materials and Methods.docx]

**Supplementary Materials and Methods**

**Histology and immunohistochemistry staining of KCs**

Liver tissue sections were repaired with EDTA (pH 8.0) and endogenous peroxidases were blocked with 3% hydrogen peroxide solution. Biopsies were performed with primary antibodies against CD68 (1:500; GB11067, Servicebio, China) incubated overnight at 4°C. Peroxidase/3, 3-diaminobenzidine (DAB^+^) was used according to the manufacturer’s protocol (ZSGB-BIO ALK Detection System Peroxidase DAB+ Rabbit/Mouse; PV-6000-D; Origene and ZSGB-BIO, Beijing, CA).

**Assay of short-chain fatty acids**

A 100-mg fecal sample was prepared with 1 mL of 50% aqueous acetonitrile. Then, 40-μL supernatants were collected after centrifugation at 14,000*g* for 10 minutes and mixed with 20 μL of 200 mM 3-dinitrophenylhydrazine (3NPH) (Sigma-Aldrich, St. Louis, MO, USA) in 50% aqueous acetonitrile and 20 μL of 120 mM N-(3-dimethylaminopropyl)-N´-ethylcarbodiimide (EDC)-6% pyridine (Sigma-Aldrich) solution in the same solvent for derivatization at 40°C for 30 minutes. To obtain isotope-labeled internal standards (IS), 50 μL of a mixed standard solution containing 4 mM of acetate, 2 mM of propionate, and 1 mM of each of the other four SCFAs (Sigma-Aldrich) were added to a mixture containing 1 mg of ^13^C6-3NPH•HCl (Quality Control Chemicals Inc., Walnut, CA, USA), 25 μL of 120 mM EDC in 50% aqueous acetonitrile, and 25 μL 6% pyridine solution in the same solvent and derivative under the same conditions mentioned previously. A mixed standard solution containing 1 mM of each SCFA was diluted to have concentrations of 0.2 μM to 500 μM, and the resulting solutions were used as working standard solutions and further derivatives (kept at 40°C for 30 minutes).

Extracts were separated by a BEH C18 column (2.1×100 mm and 1.7 μm, Waters, Manchester, UK). A binary solvent system comprising mobile phase A (consisting of 0.01% formic acid and 100% H2O) and mobile phase B (0.01% formic acid and 100% acetonitrile) was used. An 18-minute gradient with a flow rate of 350 μL/min was used as follows: 0-1.5 minutes at 5% B; 1.5-4 minutes, 5-15% B; 4-12 minutes, 15-55% B; 12-13 minutes, 55-98% B; 13-15 minutes, 98% B; and 15.1-18 minutes, 5% B. The column chamber and sample tray were kept at 40°C and 10°C, respectively.

The source parameters were as follows: spray voltage, 1000 V; ion transfer tube temperature, 350°C; vaporizer temperature, 450°C; sheath gas flow rate, 40 Arb; auxiliary gas flow rate, 20 Arb; and collision-induced dissociation gas, 2.0 mTorr.
