# Supplementary figures and images for: Fecal Microbiota of Diarrhea-Predominant Irritable Bowel Syndrome Patients Causes Hepatic Inflammation of Germ-Free Rats and Berberine Reverses It Partially
Source: Biomed Res Int. 2019 Apr 3;2019:4530203. doi: 10.1155/2019/4530203 (PMC6470425; doi:10.1155/2019/4530203)

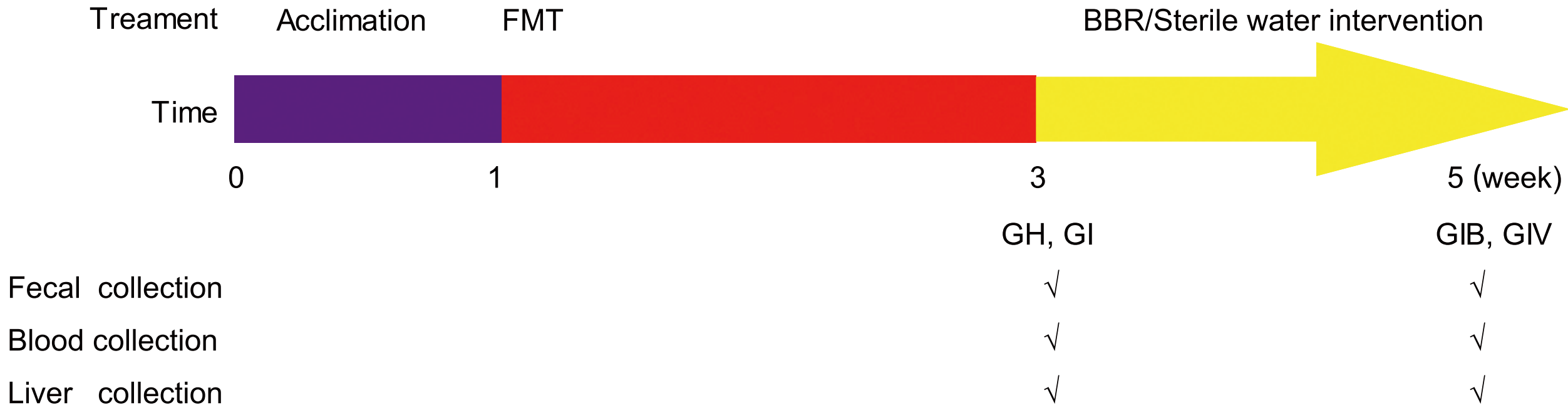

Supplement: Supplementary Materials — and Methods: histology and immunohistochemistry staining of KCs and assay of short-chain fatty acids. Supplementary Figure S1: flowchart of animal experiment. Supplementary Figure S2: inflammatory factor and serum biochemical parameters of the liver after BBR intervention. Supplementary Table S1: hepatic pathology measurements. Supplementary Table S2: clinical measurements. Supplementary Table S3: different abundance in phylum level of GH group and GI group. Supplementary Table S4: quantification of fecal SCFAs. Supplementary Tables S5 and S6: GI/GH group significant different quantified identification filtering negative ions and positive ions, respectively. Supplementary Table S7: different abundance in phylum level of GIB group and GIV group. [file 4530203.f1.zip › s/supplementary figure S1.pdf]

**a**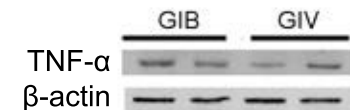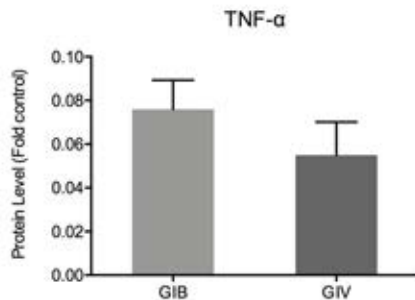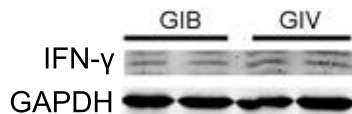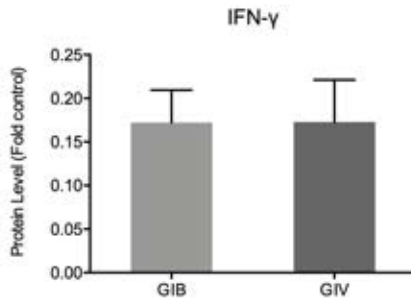**b**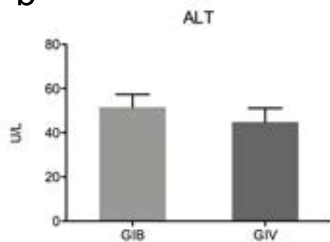**c**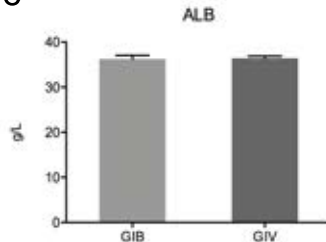**d**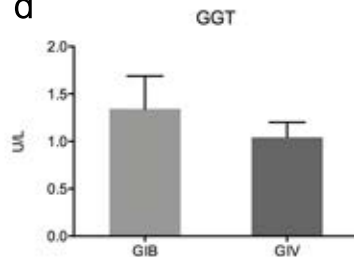

Supplement: Supplementary Materials — and Methods: histology and immunohistochemistry staining of KCs and assay of short-chain fatty acids. Supplementary Figure S1: flowchart of animal experiment. Supplementary Figure S2: inflammatory factor and serum biochemical parameters of the liver after BBR intervention. Supplementary Table S1: hepatic pathology measurements. Supplementary Table S2: clinical measurements. Supplementary Table S3: different abundance in phylum level of GH group and GI group. Supplementary Table S4: quantification of fecal SCFAs. Supplementary Tables S5 and S6: GI/GH group significant different quantified identification filtering negative ions and positive ions, respectively. Supplementary Table S7: different abundance in phylum level of GIB group and GIV group. [file 4530203.f1.zip › s/supplementary figure S2.pdf]
